# Direct Laser Writing of Copper Micropatterns from Deep Eutectic Solvents Using Pulsed near-IR Radiation

**DOI:** 10.3390/nano12071127

**Published:** 2022-03-29

**Authors:** Ekaterina A. Avilova, Evgeniia M. Khairullina, Andrey Yu. Shishov, Elizaveta A. Eltysheva, Vladimir Mikhailovskii, Dmitry A. Sinev, Ilya I. Tumkin

**Affiliations:** 1 School of Physics and Technology, ITMO University, 197101 St. Petersburg, Russia; eaavilova@itmo.ru (E.A.A.); e.a.eltysheva@itmo.ru (E.A.E.); sinev@itmo.ru (D.A.S.); 2 Institute of Chemistry, Saint Petersburg State University, 199034 St. Petersburg, Russia; e.khayrullina@spbu.ru (E.M.K.); andrey.shishov.rus@gmail.com (A.Y.S.); v.mikhailovskii@gmail.com (V.M.); 3 SCAMT Laboratory, ITMO University, 197101 St. Petersburg, Russia

**Keywords:** laser-induced metal deposition, copper, direct laser writing, LIPSS, deep eutectic solvents

## Abstract

In this study, we developed a method for the fabrication of electrically conductive copper patterns of arbitrary topology and films on dielectric substrates, by improved laser-induced synthesis from deep eutectic solvents. A significant increase in the processing efficiency was achieved by acceptor substrate pretreatment, with the laser-induced microplasma technique, using auxiliary glass substrates and optional laser post-processing of the recorded structures; thus, the proposed approach offers a complete manufacturing cycle, utilizing a single, commercially available, pulsed Yb fiber laser system. The potential implications of the presented research are amplified by the observation of laser-induced periodic surface structures (LIPSSs) that may be useful for the further tuning of tracks’ functional properties.

## 1. Introduction

Localized metallization of flexible and rigid dielectric materials is a powerful tool for surface engineering and modification. Metallic patterns are of great interest in various fields of science and technology, where they can find applications in the production of electronics, sensor devices, etc. However, the majority of well-developed and widely used patterning techniques belong to the top-down family of methods; the most striking example of them is lithography, which requires several labor-consuming stages of processing, and has a low yield of functional products, compared to the amount spent on precursors [1,2,3].

It is also worth mentioning methods of pattern fabrication, such as roll-to-roll (R2R) gravure printing [4] and copper electrodeposition [5,6]. Despite these methods offering the valuable advantage of a high production rate, these technologies, nevertheless, require expensive equipment and enormous volumes of copper plating solutions. Moreover, these methods are typically bounded by the narrow range of combinations of metals and substrates available for processing, and are not usually economically viable for the small-scale production of elements and coatings with specific properties [7].

In this regard, the development of new effective ways of fabricating metal structures is in high demand, especially for bottom-up approaches, which are more environmentally friendly and cost effective, due to the rational use of precursors and a significant decrease in the number of processing stages. One of the promising directions in this area is direct laser writing, where the availability and fairly low cost of laser equipment makes it possible to obtain all the benefits that they provide, in terms of the localized synthesis of functional materials. The main advantages of laser-assisted techniques include, in general, the extremely high levels of locality and productivity [8,9,10]. For instance, femtosecond laser sintering has been shown to be a promising method for recording copper, nickel, and cobalt conductive patterns on polymers and other types of substrates, but the high cost and instability of fs laser sources, in particular, are still the essential issues that hinder the full transition of this technique from laboratories to industry [11,12,13]. Established approaches, such as pulsed laser deposition (PLD) and laser chemical vapor deposition (LCVD), may be useful for fabricating materials with unique properties, despite the low processing speeds compared to other laser methods, and the implementation of toxic and expensive precursors [14]. The two-stage method of selective surface activation, followed by metallization, seems to be promising for industrial use [15,16]; although, currently, it is only available for the deposition of copper patterns and films.

The other member of the direct laser writing methods family is laser chemical liquid deposition (LCLD). This technique was conceived as a way of localizing copper electroless deposition, by means of initiating the chemical reduction of copper ions within the focal point of a laser beam. This method allows one to produce patterns without photomasks, out of cheap, commercially available reagents [17]. The further development of this approach has led to significant expansion of the list of materials available for space-selective deposition, including Cu, Pd, Ni, Ag, Ru, Ir, and Pt [18,19,20,21,22,23,24,25]. The deposition process works on both semiconductor and dielectric substrates, which are widely used for photonic (including metamaterials), electronic, optoelectronic, and sensoric applications [26,27,28]. However, at the same time, one of the significant drawbacks of the LCLD is the low metallization rate compared to other techniques. A promising approach to overcome this problem is the rational design of precursors, with consideration of the physical, chemical, and optical properties of lasers, substrates and solutions, instead of the system modifications used for electroless plating [29,30,31,32]. Solutions based on deep eutectic solvents (DES) is a prospective system for laser deposition. DESs are inexpensive, eco-friendly solutions, which have the ability to dissolve metal salts in high concentrations and at significantly higher boiling points than water and common organic solvents [33,34,35]. The combination of these exceptional properties is heavily exploited for the synthesis of various functional materials [36,37,38], including the laser-assisted fabrication of copper and nickel electric contacts onto glass substrates [39,40]. Regarding chemical deposition with CW lasers, DESs based on choline chloride and organic acid have dramatically accelerated scanning rates (more than two orders of magnitude), compared to the regular aqueous solution (from ~2.5 µm/s for water-based systems to ~2 mm/s for DES) [41].

This work is focused on the investigation of the laser-induced deposition of copper from deep eutectic solvents, using a nanosecond pulsed laser. Ns pulsed lasers, in comparison with continuous wave lasers, provide greater localization of the impact zone; therefore, their application for the fabrication of microelectronic elements and devices may lead to the recording resolution being decreased. Moreover, pulsed radiation offers more parameters to be controlled with high precision (such as repetition rate, pulse energy, etc.), which makes fine tuning the processing conditions possible [42,43,44,45]. The features of the deposition process and the parameters affecting its efficiency have been studied; the importance of surface pre-activation of the acceptor substrate, with the laser-induced microplasma technique [46,47], has also been highlighted and analyzed. Furthermore, new promising areas of research have been outlined, including the formation of laser-induced periodic surface structures (LIPSSs). Moreover, this work clearly demonstrated that commercially available laser systems are suitable for laser deposition from DESs, and that there is no necessity for unique and sophisticated ad hoc laboratory setups; this fact makes the proposed technique industrially valuable.

## 2. Methods and Materials

DESs, consisting of 1 g of choline chloride, 1.07 g of tartaric acid, and 2 g of copper acetate Cu(CH_3_COO)_2_, were used as a solution for laser-induced deposition. All chemical reagents in this work were of analytical grade, obtained from commercial suppliers, and used as received. Choline chloride, copper acetate, and tartaric acids were purchased from Sigma Aldrich (St. Louis, MO, USA). The detailed procedure for DESs preparation can be found elsewhere [41]. In brief, the choline chloride, tartaric acid, and copper acetate were placed in a 20 mL glass vial and heated in the drying cabinet at 120 °C, for approximately 10–15 min. Once the mixture started to liquify, the forming DES was placed in a heating magnetic stirrer at 130 °C and stirred for 40 min until complete homogeneity was achieved.

Laser-induced deposition was performed using a technological commercially available laser processing complex MiniMarker2 (Laser Center Ltd., St. Petersburg, Russia), based on the pulsed fiber Yb-laser (wavelength λ = 1070 nm, maximum average power *P* = 20 W). A comprehensive description of the experimental setup has been published elsewhere [48]; the scheme of the laser system is also presented in the Supporting Information (Figure S1). Borosilicate glass (Micromed, Observation devices LLC., St. Petersburg, Russia), with a thickness of 1.0–1.2 mm, was used both as an acceptor and as auxiliary substrates. Glass slabs were rinsed with isobutanol and water, and then structured by the laser-induced microplasma technique, using the same laser processing complex. A structured glass slab was covered with a thin (about 1–2 mm) uniform layer of DES and then with auxiliary glass, providing a “sandwich” sample (auxiliary glass–DES–acceptor glass). This sample structure kept the solution in the heated area due to surface tension, and also allowed the thickness of the solvent to be adjusted. The “sandwich” sample was placed on the coordinate table of the technological laser processing complex and processed with the laser beam focused on the solution–acceptor glass interface.

Five millimeter long linear patterns and structures of complex topology were deposited on the glass substrate to research the features of this method of deposition. Laser parameters (scanning speed V, average power *P*, and number of consequent exposures N) were varied, and the influence of acceptor substrate structuring was also studied to evaluate the effect of substrate adhesion on the copper deposition. The pulse repetition rate f and pulse duration τ were constant, and equal to 20 kHz and 200 ns, respectively. Optical microscopy (Carl Zeiss Axio Imager A1.m, Carl Zeiss Microscopy GmbH, Munich, Germany), atomic force microscopy (AFM, Hommel Werke T8000, Hommel-Etamic GmbH, Thuringia, Germany), scanning electron microscopy (SEM, Hitachi S-3400N), energy-dispersive X-ray spectroscopy (EDX-AzTec Energy 350, Oxford Instruments, Abingdon, UK), X-ray crystallography (XRD, Bruker D2 Phaser, Bruker-AXS, Karlsruhe, Germany), and the Stanford Current Meter RS570 were all used to analyze the geometrical, physical, and chemical properties of the structures. The open-source software Gwyddion was used for the LIPSSs analysis.

## 3. Results and Discussion

### 3.1. Acceptor Substrate Pre-Structuring

**Figure 1:**
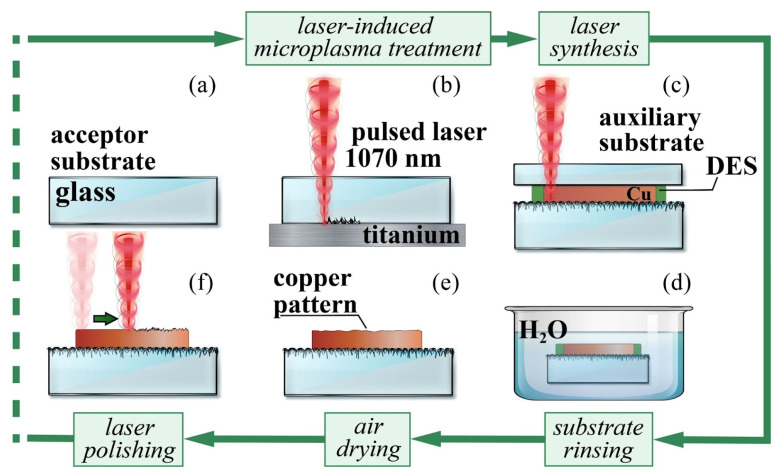
Laser-induced deposition technique. (**a**) plain glass substrate; (**b**) laser-induced micriplasma processing; (**c**) fabrication of the copper patterns under action of laser radiation; (**d**) rinsing away the leftovers of the DES layer; (**e**) air drying; (**f**) laser polishing (optional).

The substrate pretreatment procedure (Figure 1b) proved to be one of the main factors affecting the properties of metal films and tracks, including adhesion and conductivity [13,49,50]. The laser-induced synthesis with a plain glass substrate (Figure 1a) resulted in the fabrication of copper structures with poor adhesion, and with an unstable value of resistance. Changing the surface roughness was tested, and proved to be a promising and effective way to address this issue. Glass microstructuring was performed with the laser-induced microplasma method, using a commercially pure, 1 mm thick titanium plate as a target. The processing parameters for pre-structuring were as follows: average laser power *P* = 11.4 W, pulse repetition rate *f* = 99 kHz, pulse duration τ = 200 ns, scanning speed *V* = 700 mm/s, and the *y*-axis recording resolution was 40 lines per mm.

**Figure 2:**
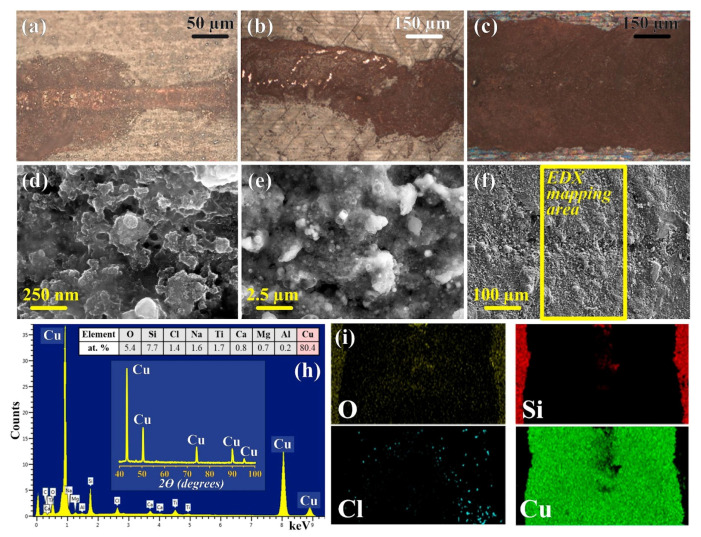
Morphology of copper tracks on (**a**) the original (non-treated substrate, type I), (**b**) laser-treated and laser-cleaned substrate (type II), and (**c**) laser-treated and non-cleaned substrate (type III). SEM images of different resolution (**d**–**f**), and XRD pattern and EDX mapping (**h**–**i**) of the track presented on the image (**c**). Laser deposition processing parameters: *f* = 20 kHz, τ = 200 ns; (**a**): *V* = 0.05 mm/s, *P* = 1.31 W; (**b**,**c**): *V* = 0.5 mm/s, *P* = 3.1 W.

The following three types of substrates were studied: original substrates (without additional processing, type I), pre-structured and laser cleaned after structuring (type II), and substrates that were pre-structured, but not cleaned (type III). The laser cleaning parameters were as follows: *V* = 700 mm/s, *f* = 99 kHz, τ = 200 ns, and *P* = 1.55 W. Deposition on type I substrates, in a wide range of experimental parameters, led to the formation of a thin, defected copper layer, without measurable conductivity (according to an on-site check, conducted with a multimeter, for the fast screening of optimal laser processing conditions). It is worth noting that the chemical reaction was only initiated at an extremely low scanning speed (less than 0.05 mm/s). In turn, significant acceleration of the deposition process was achieved by using pretreated substrates; this approach allowed us to manufacture a continuous structure, with distinctive copper-like reflectance at a scanning speed one order of magnitude higher than in the case of the type I surface (Figure 2b,c). Moreover, the residual products of the pre-structuring on the glass surface (type III) facilitated deposition, leading to an even greater amount of deposited copper (Figure 2b). This effect can be explained not only by the surface topology modification [46,47], but also by the enhanced absorption of laser radiation by the residual species, which led to more efficient heating and to the reduction of copper ions in the eutectic solvent. This assumption lies in agreement with the considerable widening of the structure, despite the increase in scanning speed (Figure 2a,b), and confirms that the main driving force of the process is thermal reduction [25], in contrast with photoinitiated deposition, using photodegradable precursors [51,52].

According to the XRD characterization, the fabricated structures consist of metallic copper. This observation was in agreement with the EDX analysis, confirming that the tracks were formed by copper, and the deviation in the Cu content, from one hundred percent on the mapping spectrum (Figure 2h,i), is attributed to the chemical elements of the substrates taken into account, unlike X-ray diffraction, due to the amorphous nature of glass. Indeed, the spot measurements of the track’s elemental composition showed pure metallic copper in most cases (Figure S2). However, the titanium signals indicate the presence of Ti species on the substrate’s surface after the pre-structuring procedure (Figure S3). Furthermore, one may notice the unevenly distributed chloride, which can be assigned to copper chloride (I) (according to previous research [39]). Their appearance was most likely caused by unsatisfactory cleaning of the samples from the reaction media. Despite the ability of the investigated DES to be washed out with water, it can be quite difficult to completely remove the reaction mixture after it has been processed with the laser, due to the high viscosity of the solutions [53], especially on the highly developed porous surface of the tracks (Figure 2d). Post processing of the copper structures could be a future research direction for the pulsed laser deposition of metals from DES; such a study may address the wide range of problems in the emerging topic of DES application in laser material science.

### 3.2. Auxiliary Substrate Usage

**Figure 3:**
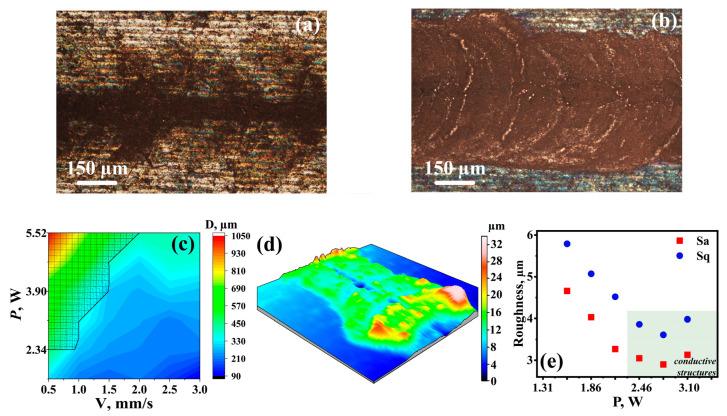
Morphology of copper tracks deposited (**a**) without and (**b**) with an auxiliary glass substrate; (**c**) processing regime diagram depending on the processing parameters, where D is the width of the structure. The area of the conductive structure’s acquisition. (**d**) Profilometry of a characteristic, electrically conductive structure. Laser parameters: *P* = 3.1 W, *V* = 0.5 mm/s, *f* = 20 kHz, and τ = 200 ns. (**e**) Surface roughness of structure depending on the laser power at *V* = 0.5 mm/s, *f* = 20 kHz, and τ = 200 ns.

One of the main issues of laser-induced synthesis from the DES is maintaining the precursor’s layer within the laser-heated area, i.e., in the reaction zone. The DESs usually have a much higher boiling point than water and common organic solvents [54], but the exact boiling temperature value highly depends on the system’s composition, and, also, in some cases, the DES decomposes before reaching the boiling temperature. Therefore, in terms of thermal stability, deep eutectic solvents offer a great advantage over classical water-based systems; however, focused laser radiation allows the reaction mixture to be locally heated up to temperatures of phase transitions. This is accompanied by an increase in excessive pressure and by a decrease in viscosity [55] in the thermally affected region. The combination of the aforementioned processes led to depletion and thinning of the DES layer, and, as a final result, to termination of the chemical reaction. The implementation of auxiliary glass was shown to be an effective strategy to fix the DES in the processing area, due to the surface tension; moreover, it allowed us to increase the uniformity of the DES layer’s thickness, as opposed to simply applying a solution on the acceptor substrate, using doctor blading. Such a “sandwich” sample architecture also prevented the interference of air bubbles and other impurities. Indeed, the auxiliary substrate promoted the fabrication of copper structures with uniform morphology (without gaps and fissures) (Figure 3a,b).

### 3.3. Laser Parameter’s Influence

Since laser-induced deposition is based on the thermally dependent reduction reaction [56], the spatiotemporal dynamics of the temperature field is the key factor determining the deposition process. The recording parameters, including scanning speed *V* and laser power *P,* influence the peak temperature, its spatial distribution, and the duration of the heating–cooling cycle.

With an increase in the scanning speed, the maximum temperature in the treatment area declined, as did the rate of the copper ion’s reduction reaction. The temperature drop can be compensated by a rise in power, so there are certain optimal processing parameters in the *P*-*V* phase space. The optimal and most productive fabrication regime was defined by the maximum value of scanning speed, which allowed us to maintain the high temperature long enough for an intensive reaction and the formation of continuous conductive patterns (Figure 3c).

An insufficient power level at a constant speed (e.g., less than 1 W at 0.5 mm/s) resulted in sluggish copper deposition and, as a consequence, the fabrication of tracks with a poor, island-like structure. An increase in laser power led to a rising width and continuity of the recorded track. Intensive metallic copper formation and the synthesis of electrically conductive structures were achieved within the optimal power range, depending on the rate of scanning speed (the shaded area in Figure 3c). Moreover, it is worth noting that operation within the optimal regime provided the deposition of tracks with lower roughness; the average copper layer thickness was about 4 μm, with random peaks up to 25 μm (Figure 3d,e). The thickness of the structure can be adjusted by post-treatment, e.g., by laser polishing, using the same setup. In addition, it is also possible to perform the laser cleaning procedure on the structures, to enhance the copper glint by removing the top layers.

The resistance of the fabricated tracks was measured by the Stanford Current Meter RS570, and conductive silver paste was used to create contact pads to minimize the influence and interference of the interface and contacts. The resistance of the 8 mm long track, fabricated in the optimal laser regime (*P* = 3.1 W, *V*` = 0.5 mm/s, *f* = 20 kHz, and τ = 200 ns), was R1 = 0.6 Ω. The increasing number of exposures at an elevated speed (*V*`` = 3 mm/s, other parameters were fixed) resulted in the deposition of a structure with R2 = 8.4 Ω. The latter observation revealed that using the multiscanning mode, with a higher speed, is not an effective way to enhance the performance of the laser deposition technique, probably due to the increase in the thickness of the structure, as well as the possible formation of defects on the initial copper layer, which led to an increase in resistance.

### 3.4. LIPSSs Formation

**Figure 4:**
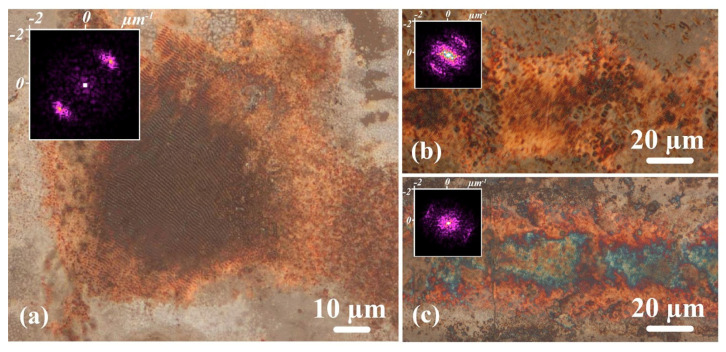
(**a**−**c**) Morphology of copper tracks recorded with LIPSSs formation. Two-dimensional FFT spectra of the areas with LIPSSs formation are shown on the insets. Recording regime *P* = 1.85 W, *V* = 0.05 mm/s, *f* = 20 kHz, τ = 200 ns, and delay duration before scanning *t* = 1 s. Structure period is 0.77 ± 0.15 μm, and the dispersion in the LIPSSs orientation angle (DLOA) is about 15−25 degrees.

The laser-induced periodic surface structure’s (LIPSSs) formation on the deposited copper layer was observed (Figure 4) in some of the processing regimes, at a scanning speed of 0.05 mm/s and a laser power of 1.3–3.1 W. LIPSSs are a commonly known phenomena, resulting from the regular polarization and wavelength-dependent patterns on the laser-treated surfaces (including copper [57,58]). Although LIPSSs have been extensively studied over the last few decades [48,59], to the best of our knowledge, this is the first report of LIPSS formation in the conditions under consideration. Nevertheless, the common model is applicable to explain LIPSS formation during the process of laser-induced deposition, using DES. The formation of an initial thin copper layer on the acceptor substrate provided the conditions for the occurrence and propagation of the surface plasmon polariton wave. This wave interfered with subsequent laser pulses, producing a regular pattern and forming LIPSSs. The regime window for recording LIPSSs is extremely narrow, and the long exposure usually used for the laser-induced deposition of copper leads to the formation of thick films, which mask the formed LIPSSs. These factors could be the reason why this effect has been overlooked previously. LIPSS formation during the process of laser-induced deposition needs further consideration, as it may lead to opportunities for the functionalization of the recorded conductive tracks, for instance, by improving their solderability [56,60] or by tuning other parameters.

## 4. Perspectives

**Figure 5:**
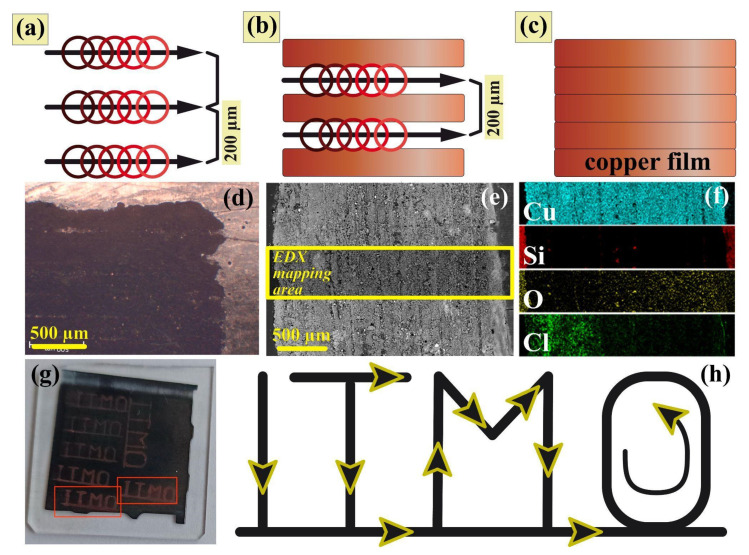
(**a**–**c**) The continuous -coating recording scheme, (**d**) optical and (**e**) scanning electron microphotography, and (**f**) EDX analysis of the recorded coating structure. (**g**) Photo and (**h**) recording scheme for the structures of arbitrary topology.

Here, we demonstrated the possibility of complex pattern recording using the laser deposition technique, as well as localized small-sized coatings. DESs exhibit quite high values of molar heat capacities [61,62], compared to water; however, at the same time, they may have rather low values of thermal conductivity [63], which can eventually lead to local overheating under the action of pulsed laser radiation. As was discussed earlier, high values of scanning speed, laser power, and pulse repetition rate were the optimal laser conditions for the fabrication of a single, electrically conductive pattern. This is because these regimes provided the necessary temperature field for the intense reduction reaction. However, when it comes to fabricating continuous films made of a group of tracks, the temperature background field after a single scan, propagating through the liquid due to thermal conductivity, leads to overheating of the treatment area during the next scan, close to the first track. Such conditions may have caused the decomposition of the solution, and the formation of defects on the acceptor substrate and on previously deposited structures. This scenario can be avoided by developing an appropriate scheme of film recording; the illustration of this is presented in Figure 5a–c. The composition of the film was confirmed by EDX (Figure 5f) and XRD analyses (Figure 2h). It consisted of pure copper and there was no significant change in the composition, compared to the single tracks; furthermore, there was no evidence of oxide formation, due to the lines overlapping and the secondary interaction with laser radiation. The film had a continuous structure, according to the low-magnification SEM images (Figure 5e). Thus, a recording scheme, with proper line spacing, makes it possible to fabricate continuous, electrically conductive films (2 mm × 5 mm), without affecting the pre-deposited tracks (Figure 5d). This finding significantly broadens the potential applications of this technique, for instance, deposits with such a morphology can be used as working, non-enzymatic electrodes for the detection of various analytes (neurotransmitters, amino acids, etc.) [19,20,60,64]. Moreover, since the recording method was based on the direct writing technique, it allowed us to fabricate conductive patterns, not only of the rectangular form, but with any arbitrary topology, as is shown in Figure 5g,h. Each component of complex structure was electrically conductive, and they were integrated into a single electrical circuit by a laser-deposited connector (solid line at the bottom of Figure 5h).

## 5. Conclusions

The fabrication of current-conducting arbitrary patterns and films, by laser-induced copper deposition, from deep eutectic solvents, using a commercially available setup, based on a ns-pulsed Yb fiber laser emitting in the near-IR range, has been shown for the first time. Preliminary structuring of the acceptor substrates, using the laser-induced microplasma technique and the addition of auxiliary substrates to keep the DES in the reaction zone, was proven to be the most effective means for the deposition of copper structures with high electrical conductivity. The resistance of the 8 mm long copper track was found to be equal to 0.6 Ω, with an average thickness of 4 µm. Furthermore, the formation of laser-induced periodic surface structures (LIPSS) in DES has been demonstrated for the first time. These findings have also outlined new directions for deeper investigations, and have demonstrated the great potential for the further development and practical application of the DES-based LCLD method.

## Data Availability

The data used or generated to support the findings of this research are included within the article and supplementary material.

## Supplementary Materials

The following supporting information can be downloaded at: https://www.mdpi.com/article/10.3390/nano12071127/s1, Figure S1: Scheme of a laser setup for laser-induced copper deposition from eutectic solvents; Figure S2: EDX spectra of copper track on laser-treated and non-cleaned substrate in three different random spots; Figure S3: EDX mapping of the manufactured pattern.
