# Supplementary material for: Direct Laser Writing of Copper Micropatterns from Deep Eutectic Solvents Using Pulsed near-IR Radiation
Source: Nanomaterials (Basel). 2022 Mar 29;12(7):1127. doi: 10.3390/nano12071127 (PMC9000477; doi:10.3390/nano12071127)
Supplement: Supplementary file 1 [file nanomaterials-12-01127-s001.zip › nanomaterials-1627737-supplementary.pdf]

nanomaterials MDPI

Supplementary material

Direct Laser Writing of Copper Micropatterns from Deep Eutectic Solvents Using Pulsed near-IR Radiation

Ekaterina A. Avilova [1], Evgeniia M. Khairullina [2,3], Andrey Yu. Shishov [2], Elizaveta A. Eltysheva [1], Vladimir Mikhailovskii [2], Dmitry A. Sinev [1] and Ilya I. Tumkin [2,*]

[1] School of Physics and Technology, ITMO University, 197101 St. Petersburg, Russia; eaavilova@itmo.ru (E.A.A.); e.a.eltysheva@itmo.ru (E.A.E.); sinev@itmo.ru (D.A.S.)
[2] Institute of Chemistry, Saint Petersburg State University, 199034 St. Petersburg, Russia; e.khayrul-lina@spbu.ru (E.M.K.);
andrey.shishov.rus@gmail.com (A.Y.S.); v.mikhailovskii@gmail.com (V.M.)
[3] SCAMT Laboratory, ITMO University, 197101 St Petersburg, Russia
* Correspondence: i.i.tumkin@spbu.ru

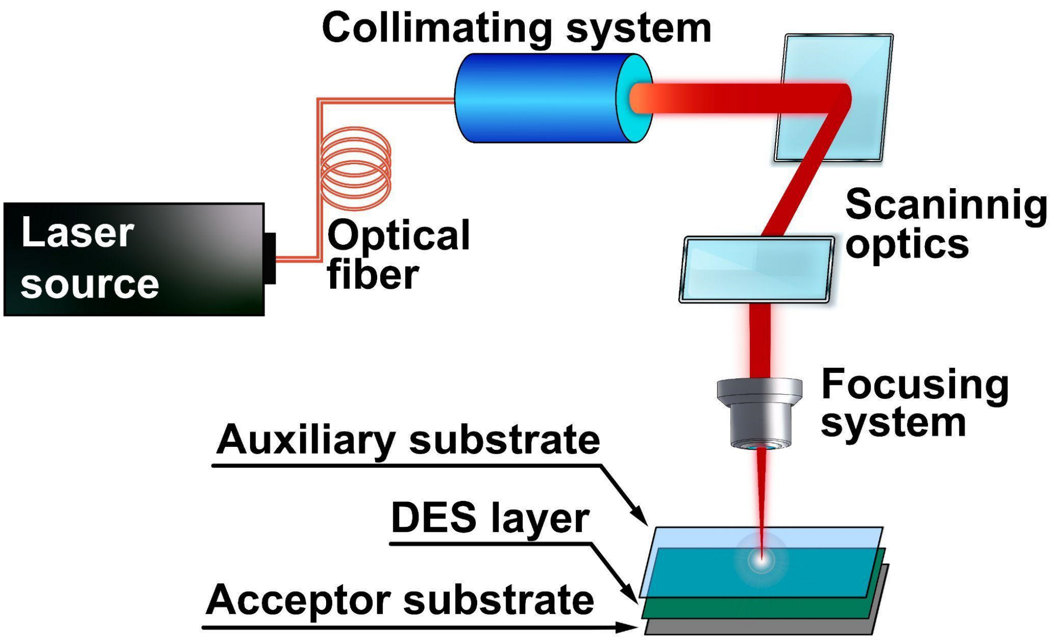

Figure S1. Scheme of a laser setup for laser-induced copper deposition from eutectic solvents.

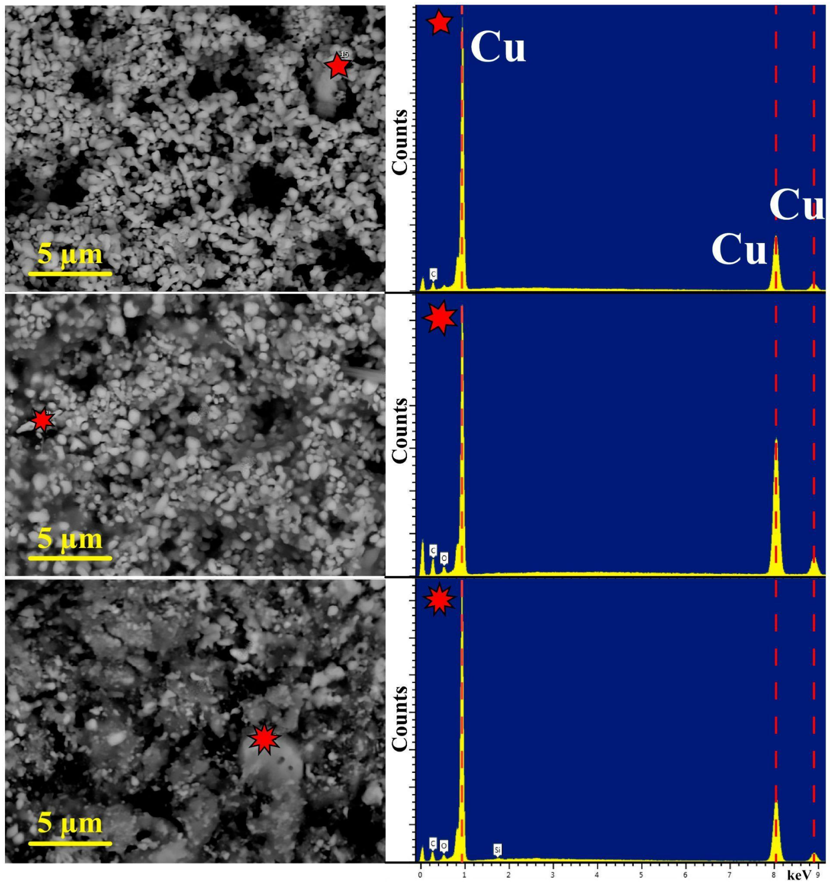

Figure S2. EDX spectra of copper track on laser-treated and non-cleaned substrate in three different random spot.

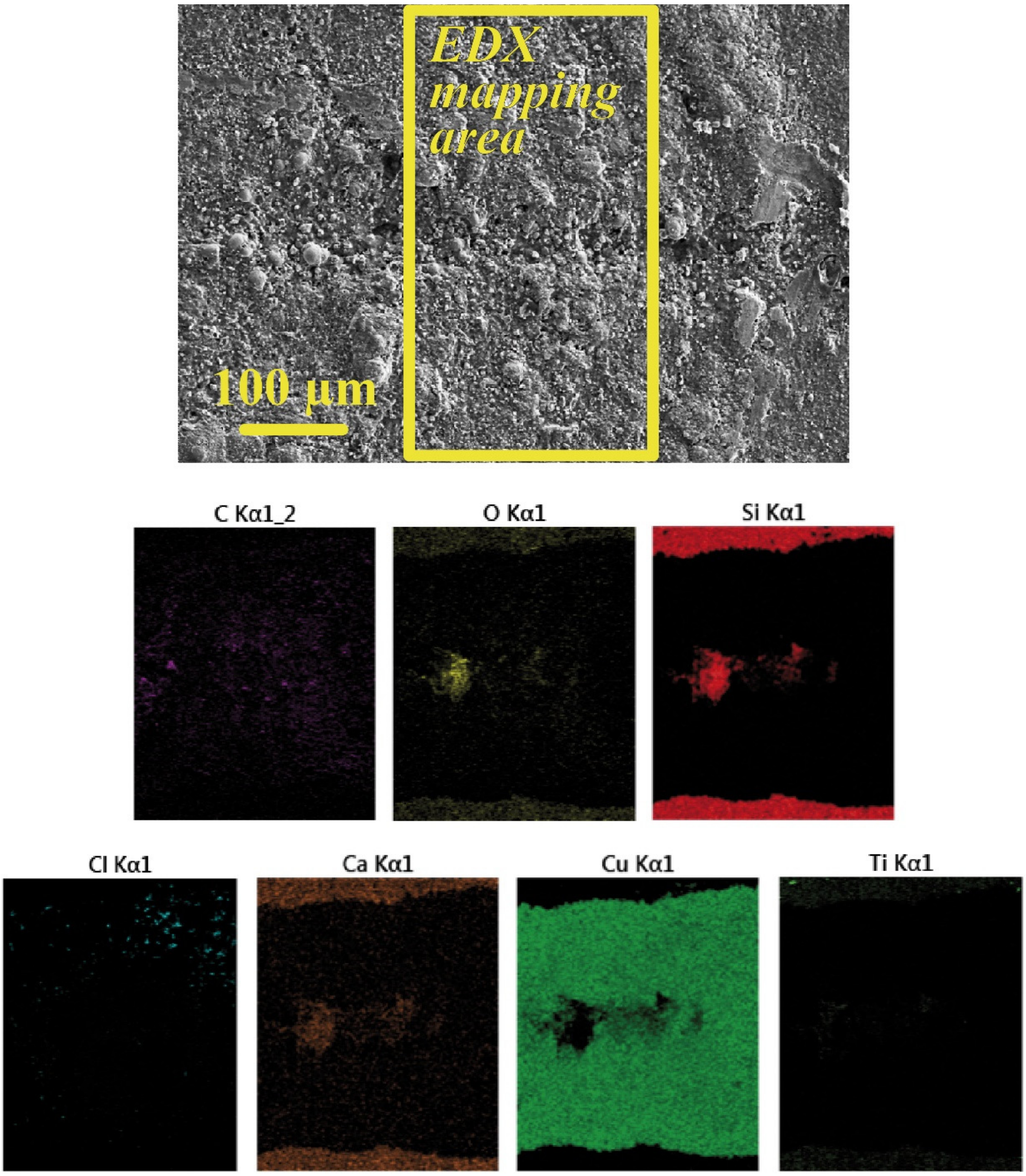

Figure S3. EDX mapping of the manufactured pattern.
